# Expression Analysis of the NLRP Gene Family Suggests a Role in Human Preimplantation Development

**DOI:** 10.1371/journal.pone.0002755

**Published:** 2008-07-23

**Authors:** Pu Zhang, Morag Dixon, Marco Zucchelli, Fredwell Hambiliki, Lev Levkov, Outi Hovatta, Juha Kere

**Affiliations:** 1 Department of Bioscience and Nutrition at Novum, Karolinska Institutet, Huddinge, Sweden; 2 Department of Clinical Science, Intervention and Technology, Division of Obstetrics and Gynecology, K57, Karolinska University Hospital Huddinge, Stockholm, Sweden; 3 Centre for Reproduction, Department of Gynecology, Uppsala University Hospital, Uppsala, Sweden; Ecole Normale Supérieure de Lyon, France

## Abstract

**Background:**

The *NLRP* (Nucleotide-binding oligomerization domain, Leucine rich Repeat and Pyrin domain containing) family, also referred to as NALP family, is well known for its roles in apoptosis and inflammation. Several *NLRP*s have been indicated as being involved in reproduction as well.

**Methodology:**

We studied, using the unique human gametes and embryo materials, the expression of the *NLRP* family in human gametes and preimplantation embryos at different developmental stages, and compared the expression levels between normal and abnormal embryos using real-time PCR.

**Principal Findings:**

Among 14 members of the *NLRP* family, twelve were detected in human oocytes and preimplantation embryos, whereas seven were detected in spermatozoa. Eight *NLRP*s (*NLRP*4, 5, 8, 9, 11, 12, 13, and 14) showed a similar expression pattern: their expression levels were high in oocytes and then decreased progressively in embryos, resulting in a very low level in day 5 embryos. However, *NLRP*2 and *NLRP*7 showed a different expression pattern: their expression decreased from oocytes to the lowest level by day 3, but increased again by day 5. The expression levels of *NLRP*5, 9, and 12 were lower in day 1 abnormal embryos but higher in day3 and day5 arrested embryos, when compared with normal embryos at the same stages. *NLRP*7 was down-regulated in day 1 and day 5 abnormal embryos but over-expressed in day3 arrested embryos.

**Conclusions:**

According to our results, different *NLRPs* possibly work in a stage-dependent manner during human preimplantation development.

## Introduction

The *NLRP* (Nucleotide-binding oligomerization domain, Leucine rich Repeat and Pyrin domain containing) family of cytoplasmic proteins comprises 14 members with similar structure. They are located in two clusters on human chromosome 11p15 (*NLRP*6, 10 and 14) and 19q 13.4 (*NLRP*2, 4, 5, 7, 8, 9, 11, 12 and 13). Most of the family members are widely expressed in various tissues and well conserved from *C. elegans*, *D. melanogaster*, rat, and mouse to human (Information recruited from Human Genome Browser Gateway: http://genome.ucsc.edu/cgi-bin/hgGateway). It has been implicated that *NLRP*s participate in apoptosis and inflammation by activation of caspases [1], [2], where *NLRP*s are more commonly referred to as NALPs [1], PANs [3] or PYPAFs [4].

Several *NLRP*s have been found to be related to reproduction. Down-regulation of *NLRP* expression is connected with oocyte aging in mice [5]. *NLRP*5 (also known as Mater) null female mice are sterile because their embryos arrest at the two-cell stage [6]. Another *NLRP* member in mice, *NLRP* iota, is also required for normal preimplantation development. Injection of siRNA against *NLRP* iota into fertilized eggs results in arrested development of embryos between 1-cell and 8-cell stages [5]. A mutation in human *NLRP*7 has been found to be associated with recurrent hydatidiform moles, spontaneous abortions, stillbirths and intrauterine growth retardation [7], [8]. Additionally, over-expression of *NLRP*7 is related to development of testicular seminomas in humans [9]. *NLRP*14 mutation was discovered in five of 157 men with azoospermaia or severe oligozoospermia [10]. Transcripts of *NLRP*5, *NLRP*8 and *NLRP*9 [11], and the protein of *NLRP*5 [12], have been detected in bovine oocytes and preimplantation embryos.

We reported earlier the expression of *NLRP*5 in human fully-grown GV oocytes [13]. Here, we studied, using the unique human gametes and embryo materials, the expression of all the 14 *NLRP* members in human gametes and preimplantation embryos at day 2 (D2), day3 (D3), day 5 (D5), and compared their expression levels between normal and abnormal embryos. According to our results, different NLRPs possibly work in a stage-dependent manner during early human development.

## Results

### Selection of *PSMB6* as an internal control gene

In order to compare gene expression levels across several developmental stages, it is essential to relate the test gene to control genes (“housekeeping genes”) that are stably expressed at the different developmental stages. We started by studying the expression of two commonly used internal control genes: beta-actin and GAPDH in human oocytes and embryos, but found that the expression levels of these two genes in human oocytes and embryos were not stable enough to act as good internal controls. The instability of beta-actin and GAPDH has also been observed in both bovine and mouse oocytes and embryos [14], [15]. Hence, we then selected housekeeping/maintenance genes from the list provided by Affymextrix. They have tested 11 different human adult and fetal tissues and found 47 transcripts expressed at the same level in all the tissues [16]. PSMB6 and EEF1A1 are listed among these 47 genes. Our microarray data (Zhang et al. unpublished) showed that both were expressed at similar levels in human oocytes and embryos. We verified the results by testing the expression of PSMB6 and EEF1A1 at five developmental stages in oocytes (GV, MI, MII) and embryos (D2, D3, D5). Real-time PCR showed the same pattern for PSMB6, but not for EEF1A1. The average Ct value of PSMB6 in individual oocytes and embryos was 29.07±0,79 (Mean±SD). PSMB6 was therefore chosen as a stable internal control in real-time PCR when human oocytes and/or embryos are used, especially when the tested genes have similar expression levels as PSMB6.

### Expression of *NLRP* family genes in normal development from oocytes to day 5 embryos

**Figure 1:**
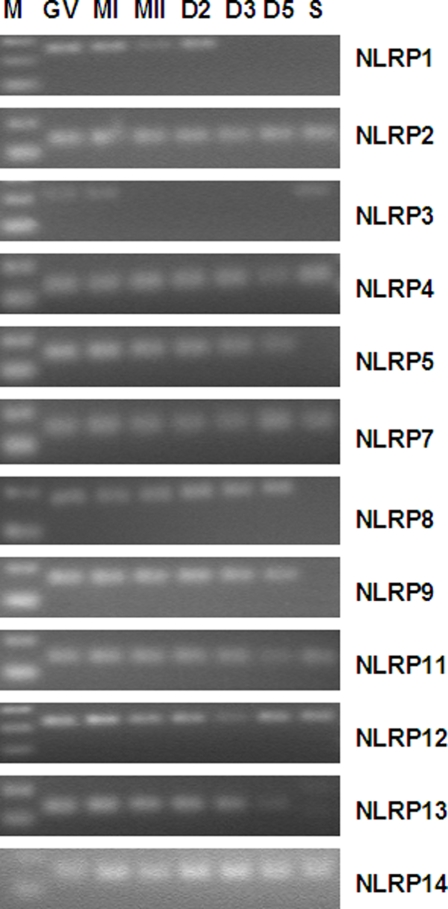
Detection of of *NLRPs* in human gametes and embryos by PCR. PCR products were about 70 bp in GV, MI, MII oocytes, day2 (D2), day 3(D3), day 5(D5) embryos and spermatozoa (S). M: marker. *NLRP1* was detected in GV, MI, MII oocytes and D2 embryos, but not in later stages. *NLRP2, 4, 7, 11, 12 and 14* were detected in all samples. *NLRP3* was only detected in GV, MI oocytes and spermatozoa. *NLRP5, 8, 9, 13* were detected in all oocytes and embryos, but not in spermatozoa. *NLPR6* was only detected in spermatozoa and *NLRP10* was not detected in any gametes or embryos (figure not shown).

We used Affymetrix arrays to monitor global gene expression during five stages of early human development (Zhang et al., unpublished). The results suggested that almost all *NLRP* family genes were expressed during development. In order to verify and extend the findings, we performed quantitative RT-PCR assays for each *NLRP* gene. The specificity of the PCR product was confirmed by both dissociation curve and gel electrophoresis. A single peak was observed in all the dissociation curves and a single band was seen on the gel at the correct product size for each gene (Fig. 1).

Among 14 members of the NLRP family, twelve were detected in human oocytes and embryos, whereas seven were detected in spermatozoa (Fig. 1). *NLRP*2, 4, 5, 7, 8, 9, 11, 12, 13, and 14 were detected in GV, MI, MII oocytes and D2, D3, and D5 embryos. *NLRP*1 was detected in GV, MI, MII oocytes and D2 embryos, but not in later stages. *NLRP*3 was only detected in GV, MI oocytes and spermatozoa. *NLRP*2, 4, 6, 7, 11, 12 and 14 were detected in spermatozoa. *NLPR* 6 was not detected in oocytes and embryos. *NLRP*10 was not detected in any gametes or embryos.

**Figure 2:**
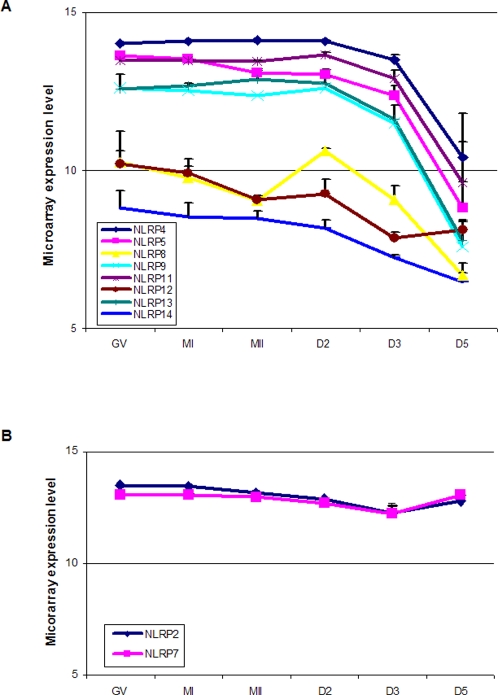
Microarray detection of *NLRPs* in human oocytes and embryos showed two different expression patterns. A: *NLRP4, 5, 8, 9, 11,12, 13 and14* were highly expressed in oocytes and then gradually decreased in embryos with a very low level in day5 (D5) embryos. B: *NLRP2* and *NLRP*7 progressively decreased from oocytes to day 3(D3) embryos then showed a sharp increase in D5. Error bars = SEM (standard error of the mean).

**Figure 3:**
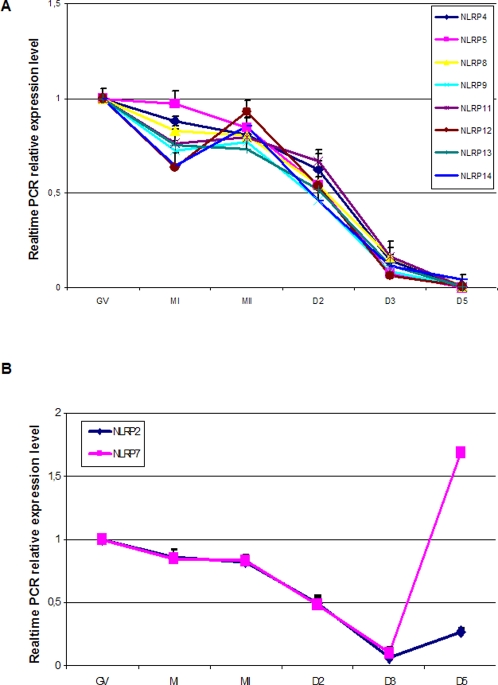
RT-PCR detection of NLRPs in human oocytes and embryos showed two expression patterns. A: *NLRP4, 5, 8, 9, 11,12, 13 and 14* were highly expressed in oocytes and then gradually decreased in embryos with a very low level in day5 (D5) embryos. B: *NLRP2* and *NLRP*7 progressively decreased from oocytes to day 3(D3) embryos, then showed a sharp increase in D5. Error bars = SEM (standard error of the mean).

Among the ten *NLRP*s (2, 4, 5, 7, 8, 9, 11, 12, 13, 14) that were detected in all the oocytes and embryos, microarrays showed two different expression patterns (Fig 2A and B). Except for *NLRP*2 and *NLRP*7, the rest were highly expressed in oocytes and then gradually decreased in embryos with a very low level in D5 embryos. The expression of *NLRP*2 and *NLRP*7 followed a similar pattern up to D3, but then showed a sharp increase in D5. These expression patterns were consistent with those revealed by real-time PCR (Fig. 3A and B).

### Expression of *NLRP*s in developmentally arrested embryos

**Figure 4:**
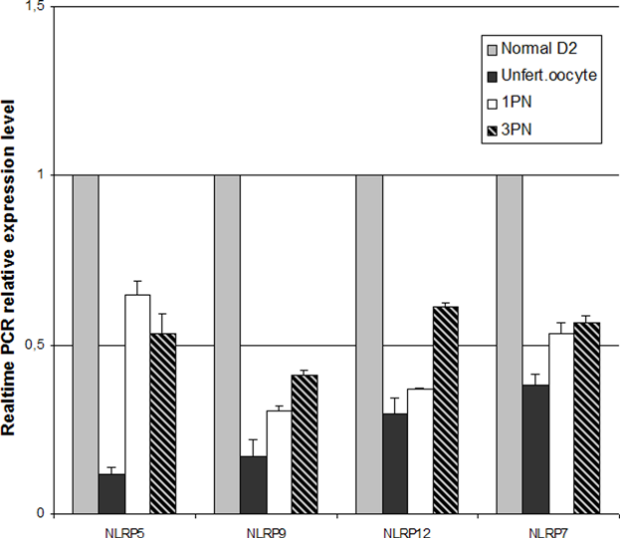
Expression of NLRP5, 9, 12, 7 in day 1 abnormal embryos, RT-PCR results. Unfert. Oocyte =  Unfertilized oocytes at day 1; 1PN =  fertilized oocyte with 1 pronucleus at day 1; (3PN) =  fertilized oocytes with 3 pronuclei at day 1. The expression levels of NLRP5, 9, 12 and 7 in day 1 abnormal embryos were lower than in normal ones. Error bars = SEM (standard error of the mean).

To figure out the possible roles of *NLRP*s in oocyte maturation, fertilization and early embryonic development, we compared the expression of *NLRP*s between normal and abnormal embryos. As we were unable to acquire normal day 1 (D1) embryos, we compared D1 abnormal embryos with normal D2 embryos (see explanation in discussion part). *NLRP*5 and *NLRP*9 showed 5 times lower expression levels in unfertilized oocytes than in normal D2 embryos, while *NLRP*12 and *NLRP*7 showed 3 times and 2.5 times lower levels respectively. In 1PN and 3PN embryos, the expression of *NLRP*9 decreased 3 times when compared with normal D2, whereas the decrease of *NLRP*5, 12, and 7 was not significant (Fig. 4).

**Figure 5:**
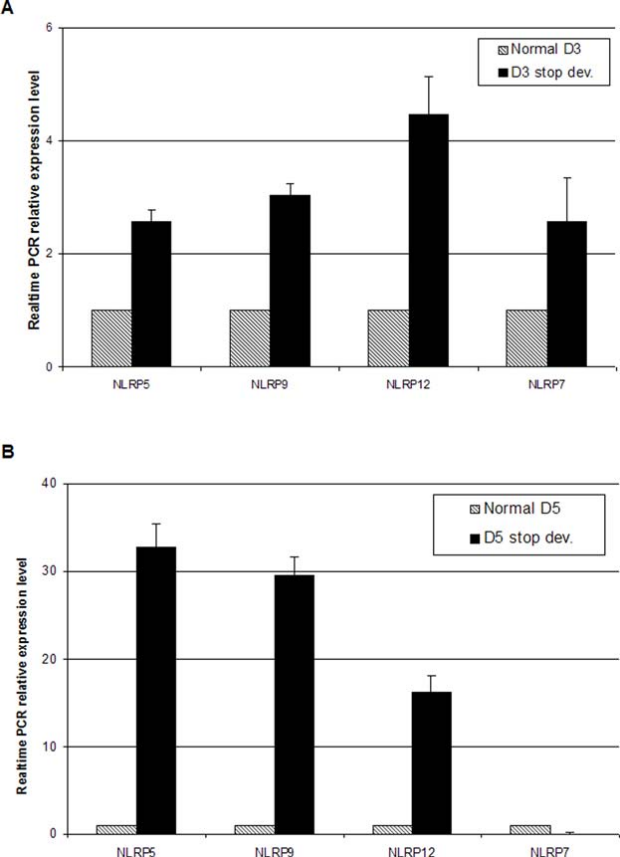
Expression of NLRP5, 9, 12, 7 in day 3 (D3) and day5 (D5) abnormal embryos. D3 stop dev. =  Embryos stop develop at D3; D5 stop dev. =  Embryos stop develop at D5. A: the expression levels of *NLRP5, 9, 12 and 7* in development arrested D3 embryos were higher than in normal D3 embryos. B: the expression levels of *NLRP5, 9 and 12* in development arrested D5 embryos were much higher than in normal D5 embryos, but the expression level of *NLRP 7* in development arrested D5 embryos was much lower than in normal ones. Error bars = SEM (standard error of the mean).

We also compared the expression levels of *NLRP*5, 9, 12 and 7 between development-arrested embryos and normal ones at D3 and D5. All four genes had a higher expression level in development-arrested embryos at D3 than in normal ones. Their expression levels increased 1.5, 2.0, 3.4 and 1.3 times respectively (Fig. 5A). In D5 development-arrested embryos, *NLRP*5, *NLRP*9 and *NLRP*12 had a higher expression level than in normal D5 embryos. The fold change was 32, 29 and 17 respectively. Oppositely, the expression of *NLRP*7 was 50 times lower in development-arrested D5 embryos (Fig. 5B).

### Phylogenetic analysis of *NLRP* genes in mammals

**Figure 6:**
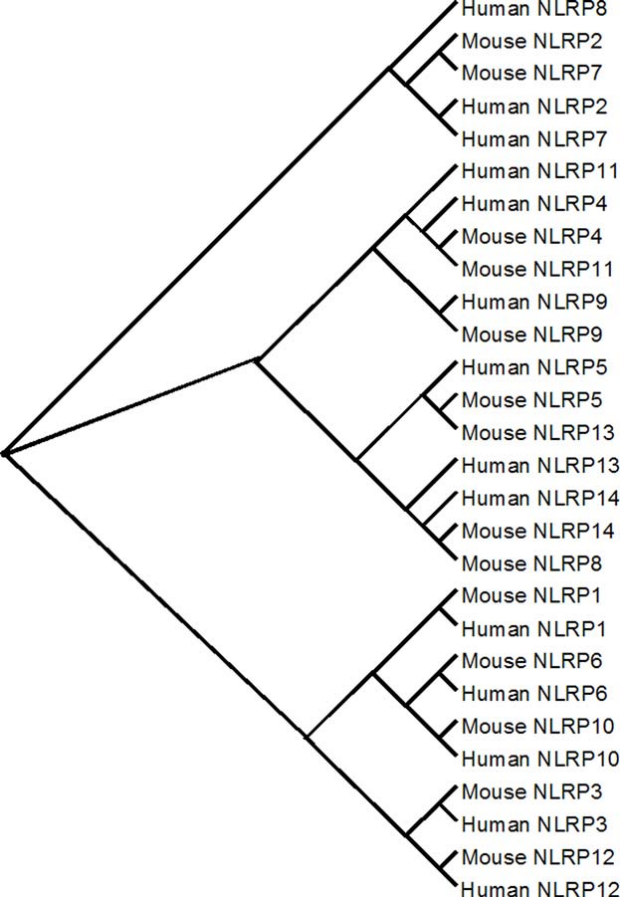
Phylogenetic analysis of the NLRP family. Homology of NLRP members between mouse and human was high, except for NLRP8. Figure showed three clusters: (1) Nalp2 and NALP7; (2) NLRP 11, 4, 9, 5, 13, 14, 8; (3) NLRP1, 6, 10, 3. The clustering of the NLRP members fits well with their expression patterns in human oocytes and embryos.

To further predict the structure and function of the *NLRP* family, we did phylogenetic analysis of the *NLRP* family using protein sequences from both mice and humans. The results (Fig. 6) showed a high homology of *NLRP* members between mouse and human, except for NLRP8. The cluster of the *NLRP* members by the phylogenetic analysis fitted well with their expression patterns in human oocytes and embryos, such as *NLRP2* and *NLRP7*. Another cluster was *NLRP1, 6, 10, 3.* All 4 of these genes were expressed at relatively low levels in oocytes and embryos. Notably, the gene closest to *NLRP5* was *NLRP13*, suggesting that *NLRP13* might have a similar function to *NLPR5*. It would be interesting to further test the function of NLRP13, both in mice and humans.

## Discussion

For the first time, we report here a comprehensive expression picture of the *NLRP* family in human gametes and preimplantation embryos. Their expression patterns were confirmed by both microarray and real-time PCR. We found downregulation of *NLRP5, 9, 12 and 7* in D1 abnormal embryos and upregulation of *NLRP5, 9, and 12* in development-arrested embryos both at D3 and D5. *NLRP*7 showed upregulation in D3 arrested embryos but downregulation in D5 when compared with normal embryos at the same stages.

It is not easy to obtain human oocytes and embryos for research, but in our large in vitro fertilisation programme, we have ethics approvals to get immature oocytes, which cannot be used for intracytoplasmic sperm injection, and mature them in vitro to metaphase II. We can also get cryo- preserved human embryos that the couples do not want to use for infertility treatment any more. Clearly abnormal embryos are also available for research in some occasions. Any functional experiments using these scarce human oocytes and embryos are not feasible. We could, however, using microarrays, real-time PCR, extensive bioinformatics methods, and comparative studies with several animal species, obtain a large amount of really new information regarding the function of these genes in human early development.

### *NLRP*5 and *NLRP*9

*NLRP*5 (Mater) is essential for mouse early embryonic development beyond the two-cell stage [6]. In mice, transcripts of *NLRP*5 are detected in oocytes from the primary follicle onwards, but they are not detectable in any stages of preimplantation embryos. *NLRP*5 protein is present until the late blastocyst stage in mice [17]. In bovine, the transcripts of *NLRP*5 have the same expression pattern as we found in humans [12]: *NLRP*5 is expressed at a high level in oocytes, and remains detectable after fertilization. It declines in preimplantation embryos and becomes hardly detectable in blastocysts.

The expression of *NLRP* 9 has been thought to be restricted to oocytes and the testis because it was not detected in somatic tissues in bovine [11], [18]. *NLRP*9 is also found exclusively expressed in oocytes in mice [19]. According to public databases, *NLRP*9 is also expressed in lung, placenta, thymus, intestine, brain and prostate. We observed that *NLRP*9 declined gradually from oocytes to blastocysts. Consistent with our study, similar expression patterns are also reported by two other studies on bovine [11], [18].

*NLRP*9 had a similar expression picture as *NLRP*5 in both normal and abnormal embryos. The decrease of *NLRP9* in D1 abnormal embryos implies its possible roles in the fertilization and zygote development; while the increase of *NLRP9* in D3, D5 arrested embryos suggests it is probably not needed in normal D3 and D5 embryos.

### *NLRP*7 and *NLRP*14

Opposite to other NLRPs, the expression of *NLRP*7 did not decrease in D5 embryos. It increased from D3 to D5, and was downregulated in developmentally-arrested D5 embryos. All these results suggest its potential role in later embryonic development in humans. This hypothesis is supported by the association of *NLRP*7 mutation with abnormal embryo development in humans [8]. The link between overexpression of *NLRP*7 and tumor development suggests that *NLRP*7 participates in cell proliferation and/ or cell differentiation. The involvement of *NLRP*7 in inflammation (where *NLRP*7 is referred to as PYPAF3) implies possible participation of *NLRP*7 in other pathways [20].

*NLRP*14 has been thought to be a testis-specific gene in humans and it might participate in inflammation as well [10]. However, mouse *NLRP*14 protein is found in oocytes at all stages of follicles, except primordial follicles; whereas no signal is detected in testis in the same study [21]. In our study, *NLRP*14 was detected in oocytes, spermatozoa and embryos. It shared a similar expression pattern with *NLRP*5 and *NLRP*9. The inconsistency in the results for *NLRP*14 could be due to species variance, or due to the diverse nominating of the same genes.

### *NLRP*12

*NLRP*12 is also known as *PYPAF7*. The expression of *NLRP*12 has been reported to be highly restricted to immune cells, there *NLRP*12 activates inflammatory signaling pathways [22], [23]. So far, there is no literature showing any relationship between *NLRP*12 and reproduction. The expression levels of *NLRP*12 in oocytes, spermatozoa and embryos were relatively low when compared to that of *NLRP*5. The expression pattern of *NLRP*12 was similar to that of *NLRP*5 and *NLRP*9, both in normal and abnormal embryos. Similar to *NLRP9*, *NLRP12* is most likely involved in the fertilization and zygote development, but in D3, D5 development.

### The indirect comparison between D1 abnormal embryos and D2 normal embryos

Although the comparison between D1 abnormal embryos and D2 normal embryos was not direct, we could still show that there was a downregulation of *NLRP*5, 7, 9 and 12 in D1 abnormal embryos. According to the overall expression patterns, D1 embryos should contain a higher level of *NLRP*s than D2 embryos. Here we saw the opposite trend instead. It suggests that the expression level of *NLRP*5, 7, and 9 in D1 abnormal embryos was much lower than in normal D1 embryos. There was a downregulation of these four *NLRP*s in D1 abnormal embryos. We can also exclude the possibility that this downregulation is due to degradation of mRNA in abnormal embryos by the observation that NLRP 5, 9,12 hade very high expression levels in D3 and D5 abnormal embryos. If there was a degradation of mRNA in abnormal embryos, the expression levels of these genes in abnormal D3 and D5 embryos would have been much lower.

Overall, the information presented in our study provides clear clues for better understanding of the roles of all *NLRP* family members in human reproduction. The changed expression of *NLRP*s in abnormal oocytes and embryos further suggests that they have potential roles in preimplantation development in humans.

## Materials and Methods

### Collection of human oocytes and embryos

This study was approved by the Ethics Committees of Karolinska Institute, Karolinska University Hospital Huddinge and Örebro University Hospital. Informed consent was obtained from all the oocyte, sperm and embryo donors. None of the donors received any financial reimbursement. The researchers of this study did not participate in obtaining consent.

Morphologically normal fully-grown germinal vesicle (GV) and metaphase I (MI) oocytes were donated by healthy women undergoing intracytoplasmic sperm injection (ICSI) treatment due to male factor infertility as described elsewhere [13]. Such oocytes cannot be injected with sperm. Metaphase II (MII) oocytes were obtained by in vitro maturation of such donated GV oocytes. The basic culture medium for oocyte maturation was Tissue Culture Medium199 (Sigma, St. Louis, MO, USA), supplemented with 10% patient‘s serum, 0.3 mM pyruvate (Sigma; St. Louis, MO), 0.075 IU/ml FSH and 0.5 IU/ml HCG (Serono, Rome, Italy).

Only embryos that could not be used in the infertility treatment were used for this study. They were from either conventional in vitro fertilization (IVF) or intracytoplasmic sperm injection (ICSI). They had been cryopreserved at the 2–8-cell stage using a three-step propanediol cryopreservation kit (Freeze kit 1; Vitrolife AB, Gothenburg, Sweden). In this study, these embryos were then thawed (Sydney IVF thawing kit, CooK IVF, Brisbane, Australia) and used at the 4-cell (D2) or 8-cell (D3) stage, or further cultured to the blastocyst stage (D5) in either BlastAssist System (Medicult, Jyllinge, Denmark) or blastocyst sequential media (Sydney IVF Blastocyst medium, CooK IVF, Brisbane, Australia).

Abnormal oocytes and embryos were collected from the clinic on the day they were to be discarded. They included unfertilized oocytes at day 1 (Unfert. oocyte), fertilized oocytes with 1 pronucleus at day 1 (1PN), fertilized oocytes with 3 pronuclei at day 1 (3PN), and embryos that stopped developing (stop dev.) at D3 and D5.

Oocytes and embryos were stored in RNAlater at −70°C. Prior to cDNA synthesis, they were thawed at room temperature in RNAlater, then moved to RNase-free PBS and washed three times before being frozen in 2 µl RNase-free H_2_O. Three to five oocytes or embryos were pooled together as one sample. Re-thawed oocytes and embryos were ready for cell-direct cDNA synthesis.

### Collection of human spermatozoa

Semen samples were donated by healthy males who underwent IVF treatment due to female infertility. Spermatozoa were obtained after standard preparation from ejaculated semen samples. Swim-up technique was used for spermatozoa preparation in all cases. Concentration and motility of spermatozoa were measured using Crismas computer spermatozoa analyzer (Image House, Denmark). Only samples with normal concentration and spermatozoa motility between 95 and 100% in final suspension were used for this study. After being washed three times in RNase-free PBS, the spermatozoa were transferred to 350 µl of RNA lysis buffer (Qiagen, Hilden, Germany) and stored at −70°C until RNA extraction.

### Real-time PCR (RT-PCR)

Materials used for real-time were different from those used in microarray. Experiments were repeated three times for each sample type using different materials.

Oocyte and embryo cDNA was synthesized using SuperScript™ III CellsDirect cDNA Synthesis system (Invitrogen, Stockholm, Sweden). Spermatozoa cDNA was synthesized with SuperScript™ III First-Strand Synthesis system for RT-PCR (Invitrogen, Stockholm, Sweden) after RNA extraction using RNeasy mini kit (Qiagen, Hilden, Germany). Oligo-dT was used for reverse transcription for all the samples. The total volume of reverse transcription is 21 µl. All the primers (Table 1) are designed by Primer Press 3.0, software for optimal primer/probe design provided by Applied Biosystems. All primer pairs span intron-exon boundaries. SYBR Green technology was applied for all the assays with ABI 7500 standard qPCR system (Applied Biosystems, Foster City, CA). The total reaction volume was 10 µl, including 5 µl 2X Power SYBR Green PCR master mix (Applied Biosystems, Warrington, UK), 0.4 µl of 5 µM forward primer, 0.4 µl of 5 µM reverse primer, 1 µl cDNA template, and 3.2 µl H_2_O. Negative controls without template were added each time. Proteasome subunit, beta type 6, also named Proteasome subunit Y (**PSMB6**), was used as an internal control. The PCR program started with 50°C, 2 minutes; 95°C, 10 minutes; followed by 40 cycles of 95°C, 15 seconds; 60°C, 50 seconds; and ended with 95°C, 15 seconds; 60°C, 1 minute; 95°C, 15 seconds. The ending step of PCR is performed to acquire the dissociation curve, validating the specificity of the PCR products. Comparative method was used for fold-change calculation. The PCR products were taken to run agarose gel (2.5%; stained with 50 _g/mL ethidium bromide). A 50 -base pair DNA ladder (Invitrogen) was used as the size marker.

**Table 1:** Primers for NLRPs used in this study

Gene symbol	Forward primer	Reverse primer
NLRP1	AAGACCAGCTGTTCTCGGAGTT	AGGCATGAGATCTCCTGGTTTC
NLRP2	TGAGGAAACCACTGTGCAACTT	AACTGAACGGAGGGATGGAA
NLRP3	GAAGAAAGATTACCGTAAGAAGTACAGAAA	CGTTTGTTGAGGCTCACACTCT
NLRP4	AACTACCCAGCAGGCAACGT	AATCAATGGGTGAGAGGTGACAA
NLRP5	CGAGGTCATGAGAGAACCATCTT	CACGCGGCGGTGAGA
NLRP6	GACCCTCAGTCTGGCCTCTGT	TCCGGCTTTGCTCTCTTCAC
NLRP7	CTTCTGTGCGGATTCTTTGTGA	TTTTTAATCTCCACTTTCTGCAGATG
NLRP8	AGGCACCCTCAGTGCAAACT	CCCGTCAAAACACCGATTAAG
NLRP9	CGCATGTGTGTGGAGAATATCTTT	CCCGCCAGTAGACGAGCTT
NLRP10	CAAGGGCTTGAAGGTCATGAA	CGCACATGCTCTCGGTATACTT
NLRP11	CGCACACTCAAGTTGTCCTATGTC	ACGAGCCAAAGCCTTGAGTAAG
NLRP12	CCAGAAACTGTGGCTGGATAGC	GCGTTGTTGGTCAGGTAAAGG
NLRP13	CTCTGAAACCACATCGTGCATT	GCAAGCAGTTGTCAGATTGCAT
NLRP14	TCAGAGGCTCGGGTTGGA	TGCAGATAAGAGCAGAGGAGAGATC

### Phylogenetic analysis of the *NLRP* family

Multiple alignment of the sequences was performed with the software ClustalX [24], while the trees where produced with TreeView 1.6.6 [25]
